# Cell Lineage of the *Ilyanassa* Embryo: Evolutionary Acceleration of Regional Differentiation during Early Development

**DOI:** 10.1371/journal.pone.0005506

**Published:** 2009-05-11

**Authors:** Morgan Q. Goulding

**Affiliations:** Section of Integrative Biology, University of Texas, Austin, Texas, United States of America; Katholieke Universiteit Leuven, Belgium

## Abstract

Cell lineage studies in mollusk embryos have documented numerous variations on the lophotrochozoan theme of spiral cleavage. In the experimentally tractable embryo of the mud snail *Ilyanassa,* cell lineage has previously been described only up to the 29-cell stage. Here I provide a chronology of cell divisions in *Ilyanassa* to the stage of 84 cells (about 16 hours after first cleavage at 23°C), and show spatial arrangements of identified nuclei at stages ranging from 27 to 84 cells. During this period the spiral cleavage pattern gives way to a bilaterally symmetric, dorsoventrally polarized pattern of mitotic timing and geometry. At the same time, the mesentoblast cell 4d rapidly proliferates to form twelve cells lying deep to the dorsal ectoderm. The onset of epiboly coincides with a period of mitotic quiescence throughout the ectoderm. As in other gastropod embryos, cell cycle lengths vary widely and predictably according to cell identity, and many of the longest cell cycles occur in small daughters of highly asymmetric divisions. While *Ilyanassa* shares many features of embryonic cell lineage with two other caenogastropod genera, *Crepidula* and *Bithynia,* it is distinguished by a general tendency toward earlier and more pronounced diversification of cell division pattern along axes of later differential growth.

## Introduction

Spiral cleavage is a form of early embryonic development that occurs widely within the lophotrochozoan superphylum [1]. Remarkably, ‘spiralian’ taxa have evolved disparate adult body plans, yet retain a close correspondence between early embryonic cell lineage (spatially defined with respect to the egg's primary animal-vegetal (AV) axis) and cell fate (i.e., clonal distribution among juvenile/larval body regions and tissues) [2]–[4]. Although spiral cleavage has been modified beyond recognition in some clades, most known mollusks, annelids, nemerteans, and polyclad flatworms show only subtle deviations from a generalized spiral cleavage pattern.

**Figure 1:**
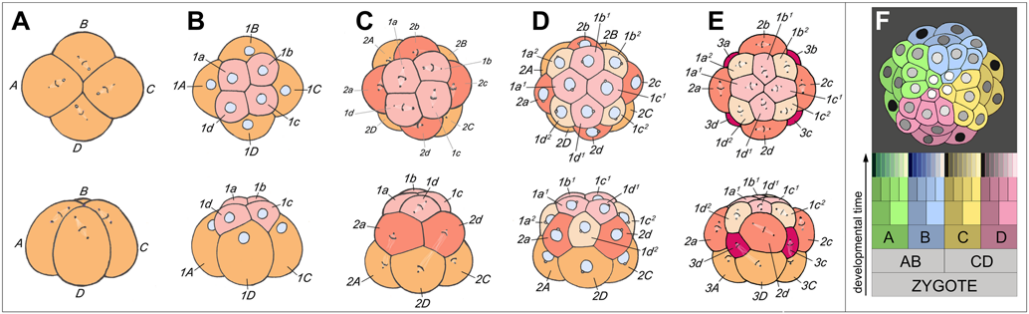
Spiral cleavage: elaborating fourfold rotational symmetry about the primary axis of polarity. (A) through (E) show the third through fifth rounds of cleavage in the basal gastropod *Trochus*, as seen from the animal pole (top row) and from one side (bottom row). (A) Mitotic spindles in the quadrant founder cells are skewed clockwise, preparatory to dexiotropic division. (B) Eight-cell interphase; the first-quartet micromeres (1a–1d) are nestled between neighboring macromeres (1A–1D). (C) Formation of the second micromere quartet by laeotropic division; the first-quartet cells in this species also divide laeotropically, but with a slight delay (a much longer delay occurs in *Ilyanassa*). (D) Sixteen-cell stage: interphase cells are nestled with new neighbors. (D) Fifth cleavage: the third-quartet micromeres are formed by dexiotropic division of macromeres; cells of both the first and second micromere quartets divide also dexiotropically, but with a slight delay. (F) The distribution of quadrants at a later cleavage stage of this relatively simple spiralian embryo. In this animal pole view, the four quadrant lineages are color-coded. First-quartet clones are shaded light, and second-quartet clones dark (After Robert, 1902). The schematic cell lineage table at bottom shows the typical relationships of the quadrants, and indicates the parallel generation of micromeres and micromere sublineages in the four quadrants of a generalized spiralian embryo.

The term ‘spiral cleavage’ reflects a characteristic spatial pattern of cell division exhibited most conspicuously from the third through fifth cell cycles. Unlike other animal embryos, spiralian early cleavage planes are never perpendicular to the AV axis. Thus, while the third cleavage in a frog, sea urchin or jellyfish embryo separates four animal (‘northern’) cells from vegetal (‘southern’) sister cells, third cleavage in a spiralian embryo separates four ‘northwestern’ cells from ‘southeastern’ sisters (the reverse chirality is also observed in some taxa) (**Figure 1A,B**). The four (typically smaller) cells around the animal pole are called micromeres, and their vegetal sisters are called macromeres. The macromeres undergo two more rounds of concerted asymmetric division, budding two more quartets of micromeres toward the animal pole (**Figure 1C–E**). The chirality of the oblique macromere divisions alternates with each cell cycle: e.g., the first quartet is budded to the northwest, the second to the northeast, and the third to the northwest once more. The micromeres themselves continue to follow the rule of alternating division chirality for one or more cell cycles. Through synchronous reiteration of obliquely oriented divisions, four founder cells thus give rise to lineages (A, B, C, D) that represent ‘quadrants’ distributed in a rotationally symmetric pattern about the AV axis (**Figure 1F**). Owing to the practically identical cell division patterns in the four quadrant lineages, early cleavage generates multiple cell ‘tiers’, or sets of four synchronously formed and phenotypically similar cells that are radially disposed about the AV axis. Most spiralian embryos exhibit tier-specific patterns of cell division at early stages; the significance of this mitotic asynchrony is unknown, but presumably reflects differential inheritance of factors regulating cell division. Indeed, in 24-cell embryos of the marine snail *Ilyanassa,* each tier is distinguished by inheriting a unique set of cytoplasmic RNA species [5].

**Figure 2:**
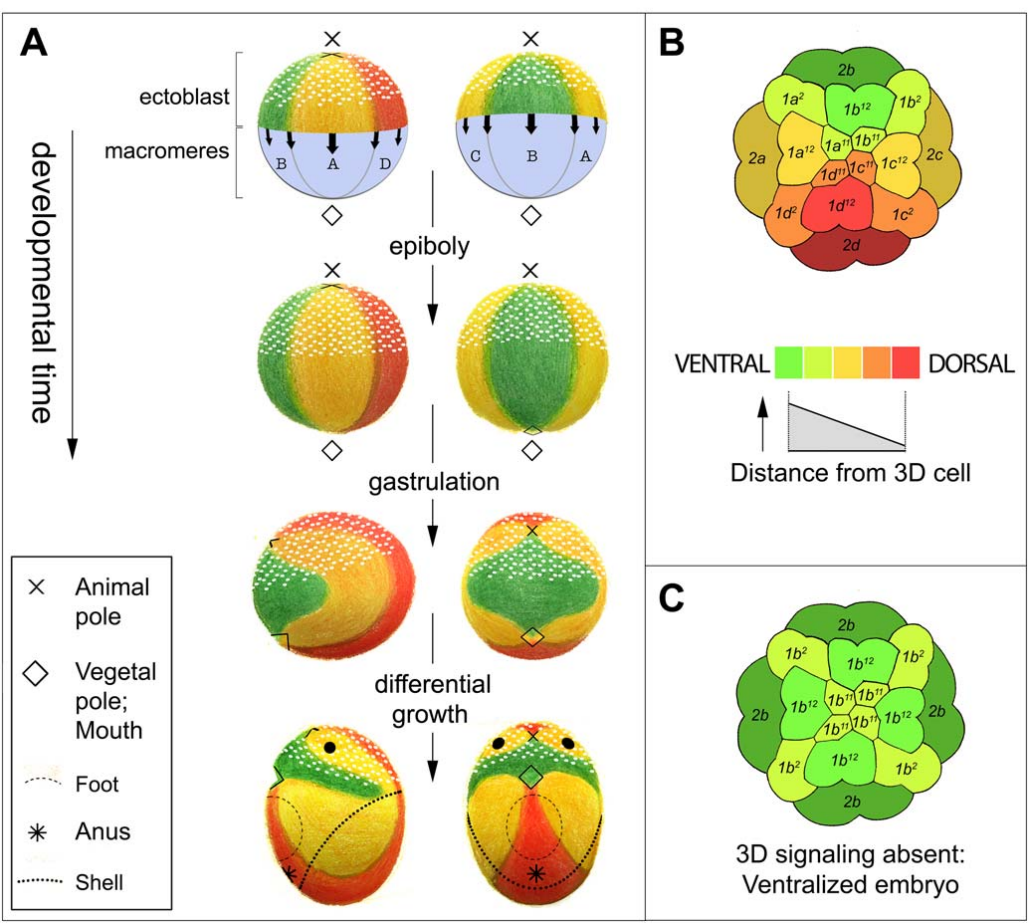
Morphogenesis and the secondary axis of the spiralian embryo. (A) Morphogenetic transformation of the rotationally symmetric spiralian embryo to a dorsoventrally polarized juvenile animal, as inferred from fate-mapping and other descriptive studies. Dorsal, lateral, and ventral ectodermal territories (these terms being adopted here to describe the embryonic secondary axis) are respectively colored red, yellow, and green. First-quartet micromere derivatives are stippled white. The ectoderm originates as a roughly hemispherical cap of micromeres that sit atop the macromeres (top row); this cap spreads by epiboly over the four macromeres until its edges converge at the vegetal pole. The creature cartooned at bottom is a schematized gastropod larva, omitting for simplicity the convoluted velum and mantle cavity as well as the overt bilateral asymmetry of the shell-forming dorsal trunk region. (B) Diagram illustrating a current model of fate specification in the early embryo: the fate of each micromere (or micromere daughter) is encoded combinatorially by its tier identity (labeled and shaded) and its position along the secondary axis (indicated by hue as in (A)). (C) Schematic of ventralized embryo as observed when the 3D signal is blocked. By default, each cell follows the tier-specific program appropriate for the B quadrant; ventral and ventrolateral fates are color-coded as in previous panels.

For the most part, each micromere clone in a spiralian embryo remains largely cohesive throughout embryogenesis, and adopts a highly predictable fate [2]. The entire ectoderm arises from the first, second and third micromere quartets (collectively, the ‘ectoblast’). **Figure 2A** shows a schematic summary of ectodermal morphogenesis, as inferred from fate mapping and other descriptive studies of embryogenesis in gastropod mollusks [6]–[11]; this scheme can be generalized in broad outline to other spiralians, though some details differ between higher-order taxa [2]–[4]. At the animal pole, the four-way junction of the first-quartet micromere clones persists to form the anterior apex of the juvenile animal. Oppositely, the vegetal extremities of the ectoblast (second and third quartet clones) converge at the vegetal pole to form the rudiment of the mouth; in many taxa (including *Ilyanassa*) this convergence occurs by epiboly, as illustrated in Figure 2A. During subsequent development, the AV axis bends extensively, displacing the mouth-forming vegetal cell group along one meridian toward the animal pole. The ectodermal territory centered on this meridian becomes shorter and wider as micromere clones spread bilaterally and/or become partially internalized; at the same time, the ectodermal region opposite to this territory elongates meridionally to form most of the trunk, including both the definitive dorsal and ventral ectoderm. In gastropods (as in most spiralians), the first-quartet micromere clones collectively form the animal's head; the boundary between the first-quartet and second-quartet clones forms the velum, a band of ciliated cells that functions in larval locomotion and feeding.

The above described transformation of the spiral-cleaving embryo into a dorsoventrally polarized animal depends on an early intercellular signal that patterns the ectoblast along a secondary axis, orthogonal to the original (AV) axis of polarity. This secondary axis is commonly designated as ‘dorsoventral’ (a shorthand term whose inaccuracy is illuminated by **Figure 2A**). In gastropods, dorsoventral ectoblast patterning depends on one of the vegetal macromeres, 3D (and/or its daughter cell 4d) [12]–[17]. A substantial body of evidence suggests that the developmental program of each ectoblast micromere (or micromere daughter) is encoded combinatorially by its quartet/tier identity and its position relative to 3D/4d (reviewed in [16]). (**Figure 2B**). Consistent with this model, each micromere quartet includes at least one early-born bilateral pair of cells that form bilaterally paired regions of the larval ectoderm [6]–[10]. Ablation of any single micromere yields a defect corresponding largely to its fate, indicating early restriction of developmental pathways [18]–[20]. Single micromeres heterotopically transplanted prior to 3D formation develop according to tier identity and position [21]. Blocking the 3D/4d signal radializes morphogenesis, with each cell adopting a default ventral fate characteristic of its tier (**Figure 2C**); in this situation, the embryo's original four-fold rotational symmetry is maintained through gastrulation and larval organogenesis [12], [17], [22]. Differential specification of micromere lineages along the dorsoventral axis is correlated with early divergence of cell division rate and geometry [12]. This early mitotic differentiation is the first morphological sign of dorsoventral pattern in the ectoderm.

**Figure 3:**
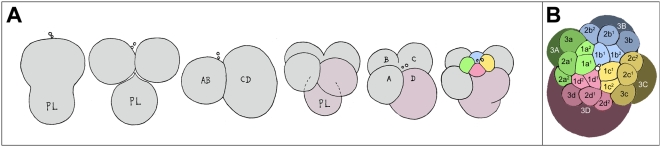
Early cleavage in *Ilyanassa obsoleta.* (A) The first three cleavages; animal pole is at top. Polar lobes forming with first and second cleavage are indicated (PL). The D quadrant founder cell and 1D macromere are shaded pink-gray. The first-quartet micromeres are color-coded by quadrant as in Figure 1F. (After Clement, 1952.) (B) 24-cell stage as seen from the animal pole (enlarged from the scale of (A) to show detail). Micromeres are color-coded by quadrant. (After Craig and Morrill, 1986.)

Our present understanding of spiralian cell fate specification owes much to studies of the mud snail *Ilyanassa*. In this species, the secondary axis is determined through an unusual mechanism: whereas in other gastropod taxa the 3D cell is specified inductively among initially equivalent macromeres, the 3D precursor in *Ilyanassa* is specified as the D cell by asymmetric segregation of vegetal cytoplasm during the first two cleavages [23] (**Figure 3A**). This segregation allows 1d, the first-quartet micromere of the D quadrant, to differ at birth from the three other first-quartet cells [12], [21]. In other respects, the ectoblast of *Ilyanassa* displays the same rotational symmetry seen in other gastropod embryos (**Figure 3B**), and a molecular marker of signaling from the D quadrant is not expressed until after the birth of the third quartet [16], [24]. As in other mollusks, the signaling 3D cell is mother to the mesentoblast 4d, which marks the definitive median plane at the 28-cell stage, and which gives rise to a bilateral pair of mesodermal and endodermal stem cell lineages [25]. Subsequent cell divisions among the ectodermal micromere lineages have not been systematically described.

In this paper I follow the *Ilyanassa* embryo through its next fifty-six cell divisions, describing the spatiotemporal division pattern from first cleavage through the eighty-four-cell stage. This analysis reveals progressive differentiation of division patterns within cell tiers, especially along the secondary axis. Such within-tier differentiation is more widespread, and also tends to be more pronounced, in *Ilyanassa* compared to the related caenogastropod genera *Crepidula* [6], [11], [26] and *Bithynia* [27]. Although the D lineage is specified at later stages in these two genera, most of the accelerated regional differentiation in *Ilyanassa* occurs after 3D/4d signaling, and thus does not depend on early D specification as a logical precondition. Moreover, the global acceleration of differential growth in *Ilyanassa* is generated by a wide variety of changes in division geometry and timing among different micromere sublineages, implying the concerted evolution of diverse factors controlling cell division.

## Materials and Methods

### Animals, Microscopy, and Data Acquisition

Snails were obtained from the Marine Biological Laboratories at Woods Hole in the winter and early spring. Snail and embryo cultures were maintained as described by Collier [28]. Eight clutches of eggs were used for this study; each cell division was examined in 3–8 embryos, in most cases representing at least two different clutches. Developmental ages were recorded with reference to the onset of first, second, third or fifth cleavage, all of which occur with predictable relative timing. A small minority of embryos which initiated the reference cleavage outside of a ten-minute median time window were removed and cultured 5–6 days alongside controls; in every case, over 90% of both groups developed formed normal veliger larvae.

Descapsulated embryos were cultured at air-conditioned room temperature of 23±1°C in 108% Jamarin U artificial seawater (JSW; Jamarin Laboratories, Japan) that was 0.45 µm filtered, pasteurized, cooled and aerated by shaking. At 15- or 20-minute intervals during the desired time window, samples of 2–5 embryos were fixed for an hour at room temperature in 90% JSW containing 2.5% paraformaldehyde and 0.1% Tween-20. Fixed embryos were washed in water, incubated in methanol for 15–30 minutes, gradually rehydrated, and stained overnight at 4°C in 1 µg/ml Hoechst 33528 and 0.1% Tween-20. After another wash, they were cleared in glycerol and mounted under clay-feet-supported coverslips in 80% glycerol, 4% n-propyl gallate, 20 mM Tris pH 9. With this mounting method, embryos could be rolled around to permit observation from different sides. Embryos were observed with epifluorescence optics under UV illumination on a Leitz compound microscope with a 25× or 40× objective. Images were acquired by a ‘video camera lucida’ method in which fluorescence was imaged with a video camera, and the outlines of nuclei were traced onto transparent plastic sheets fixed to the video monitor. All cells were identified in each fixed embryo. The timing of each cell's division was estimated as roughly the mean age of embryos in which the cell was observed in metaphase, anaphase or telophase.

### Nomenclature

Most early cell divisions in *Ilyanassa* are oblique to the animal-vegetal axis. Such a division is described as *dexiotropic* if the clockwise daughter cell (in animal pole view) is the closer to the animal pole; in a *laeotropic* division the counterclockwise cell is closer to the animal pole. Some divisions are not oblique to the AV axis; these divisions are either *transverse* (spindle parallel to the embryo's equatorial plane) or *longitudinal* (spindle perpendicular to equator). Most cells are named following convention [6], [29], [30]. Micromere-derived cells are named according to their previous lineage history (encapsulated in the name of their immediate progenitor), and the relative positioning of sister cells along the egg axis. The upper sister (closer to the animal pole) is always given a terminal superscript 1, and the lower sister a terminal superscript 2. Where a transverse division places sister cells at the same level of the egg axis, they are usually numbered with reference to the chirality of the previous division: if the mother cell formed by a dexiotropic division, then the clockwise product of this cell's next division is given a superscript 2 and the counterclockwise product a superscript 1. Following Conklin [6], this nomenclature is replaced in the case of the third-quartet derivatives with terms relating cell identity to the secondary axis. In general, when referring collectively to cells formed by analogous divisions in two or three quadrants, I use terms such as 1abc^1^; this example signifies the three upper cells formed by the division of 1a, 1b, and 1c. Following Costello [31], analogous cells in all four quadrants are referred to collectively using “m” to denote micromeres and “M” to denote macromeres (1m^1^, for example, is used instead of 1abcd^1^). Finally, Conklin [6] named derivatives of the mesentoblast 4d on the basis of their supposed fates in *Crepidula*; these fates need to be confirmed using modern methods, and 4d derivatives are renamed here on the basis of lineal relationships and the positioning of sister cell nuclei [25]. As the 4d lineage seems to undergo identical division patterns in *Crepidula* and *Ilyanassa,* it is easy to refer back to the original cell names. Throughout this paper the secondary axis of the embryo will be called ‘dorsoventral’ according to a current, arbitrary convention; it should be noted that this axis does not correspond by cell lineage distribution to any definitive anatomical axis (**Figure 2A**). The ‘dorsal’ pole of the early embryo's secondary axis is marked by the center of the bilaterally symmetric 4d clone. Cells that lie on dorsal, ventral, left or right meridians are said to be positioned radially, while cells positioned forty-five degrees away from these meridians are positioned interradially.

## Results

### First two cleavages and polar lobe segregation

**Figure 4:**
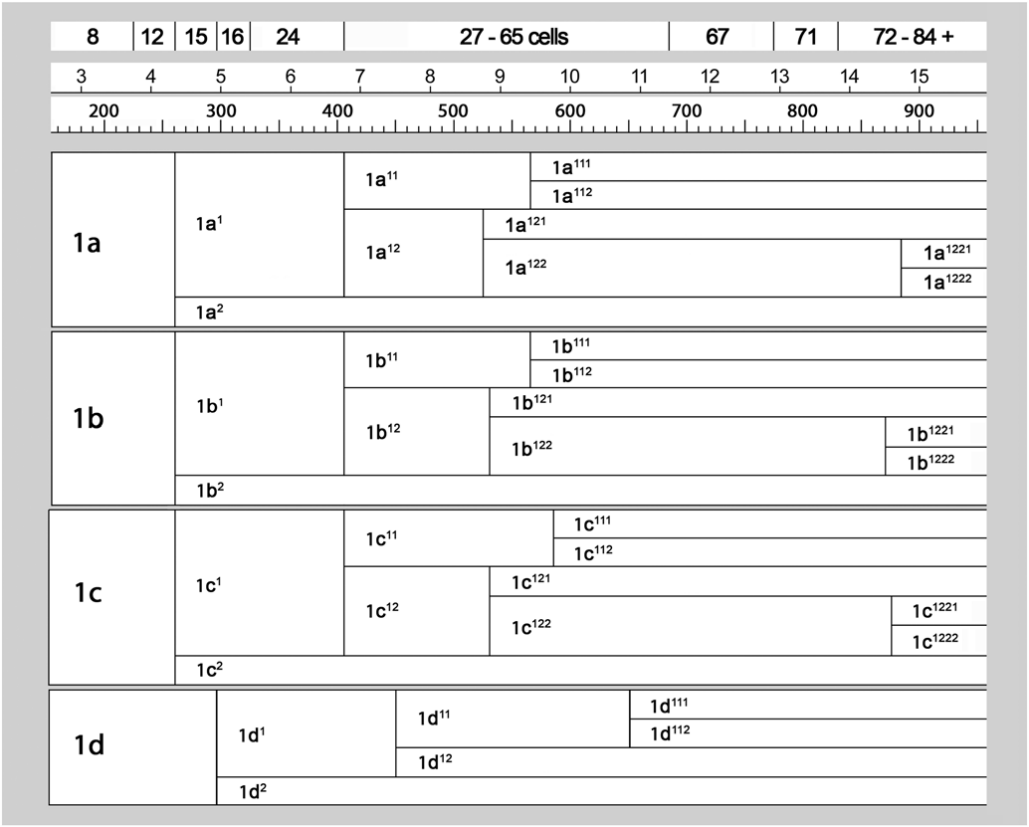
Cell lineage table of the first micromere quartet.

Early cleavage in *Ilyanassa* has previously been described [12], [32], and is summarized in **Figure 3A**. Like the egg's two meiotic divisions, first cleavage is accompanied by polar lobe formation [33], [34]. The first cleavage furrow initiates at the animal pole and progresses vegetally in a roughly meridional plane. The polar lobe relaxes into the CD cell, which then forms another polar lobe at second cleavage, shunting it to its western daughter cell, D. Second cleavage is slightly asynchronous, CD entering mitosis before AB [32]. As in the first cleavage, AB and CD form meridional cleavage furrows that progress from animal to vegetal. The planes of second cleavage are almost orthogonal to the first cleavage plane, so the animal-pole surfaces of the four quadrant-founder cells (A, B, C, D) end up bordering each other in a regular fashion, close to the site of polar body formation. A slight laeotropic skew between the AB and CD cleavage planes place the A and C cells visibly higher on the AV axis compared to B and D; this geometry is common among gastropods and many other spiralians, and presages the overt dexiotropic chirality of the following cleavage. The interval between first and second cleavage lasts about 75–80 minutes. In subsequent generations, the same cell cycle length is observed in the quadrant founder cells (M) and in their macromere daughters (1M); all other cell cycles, with the exception of some cells in the 4d lineage, are longer. Temporal cell division patterns in the four micromere quartets are charted in **Figures 4** **,** **5** **,** **6** **,** **7**.

**Figure 5:**
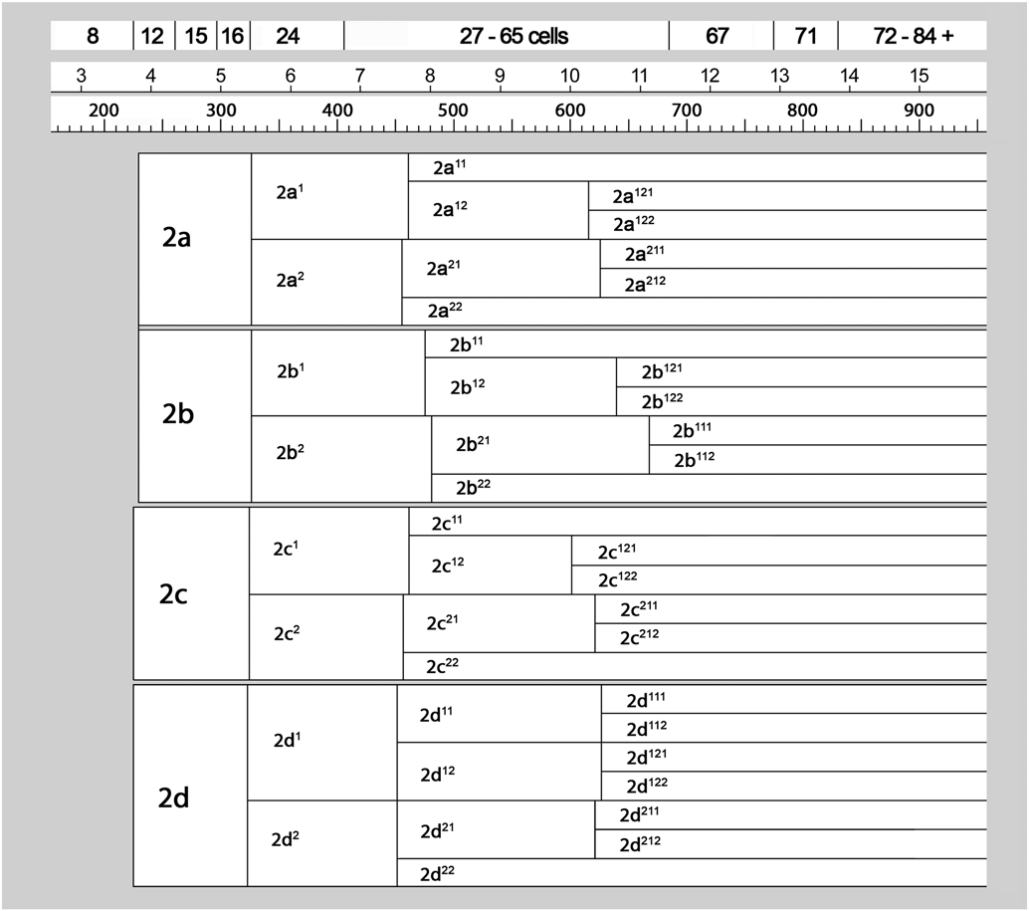
Cell lineage table of the second micromere quartet.

**Figure 6:**
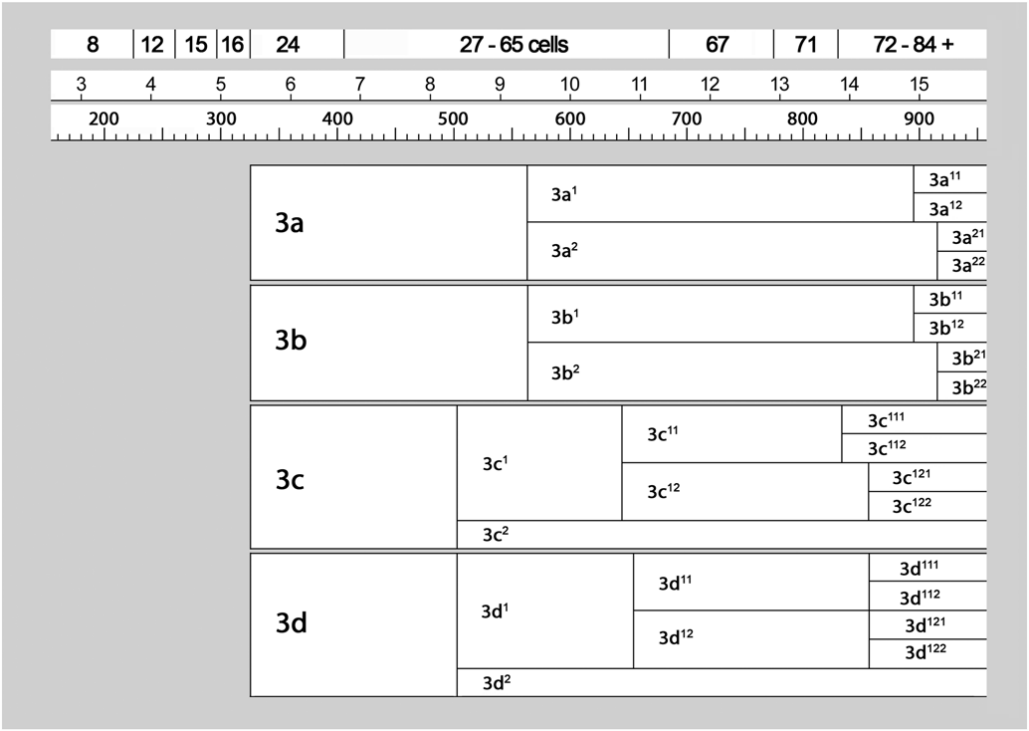
Cell lineage table of the third micromere quartet.

**Figure 7:**
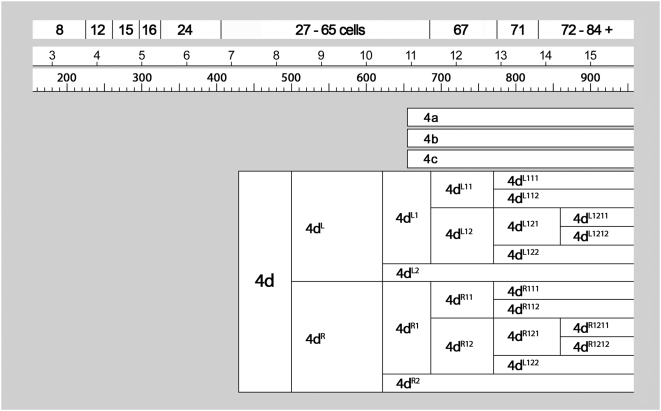
Cell lineage table of the fourth micromere quartet.

### First quartet (Figure 4)

At third cleavage, the quadrant founder cells divide asymmetrically to form the first quartet micromeres around the animal pole (**Figure 3A**). Mitotic spindles form synchronously in C and D, with A and B together lagging a few minutes behind. In spite of the apparent mitotic synchrony, the D cell consistently begins cytokinesis slightly before C. The dorsal micromere 1d is significantly smaller at birth than 1a, 1b and 1c [12], [35].

The first quartet cells are immediately distinguished from their macromere sisters by a retarded division schedule: the 1a, 1b, and 1c cells divide about 30 minutes after the macromeres (105 minutes after their formation), and 1d divides after an additional delay of 30–35 minutes. The division of each micromere is laeotropic and unequal, separating a large cell (1m^1^) at the embryo's apex from a much smaller ‘turret cell’ (1m^2^) distal to the animal pole. Following cytokinesis, the nuclei of the 1m^1^ cells grow as big as the nuclei in their mother cells, while the turret cell nuclei remain very small and condensed. The turret cells typically remain in interphase through the next eleven hours of development at least.

**Figure 8:**
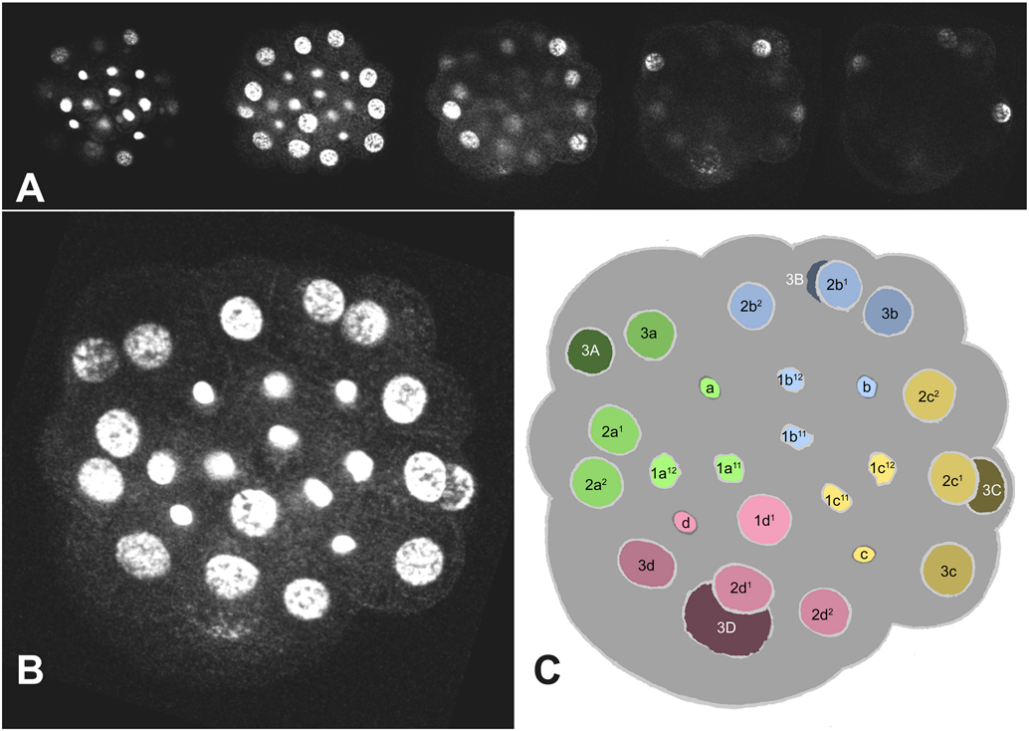
27-cell embryo, 410 minutes. Confocal images (A) are shown in a z-projection (B) and an interpretive tracing (C). The 1abc^1^ cells have just divided and nuclei are in telophase; 1d^1^ is still in interphase. 3D is in prophase. The drawings in Figures 8–21 are from camera lucida tracings (see Material and Methods). Most embryos are seen approximately from the animal pole, with the secondary axis oriented more or less vertically. Those in Figures 17, 18 and 21 are seen from more or less dorsal aspects. All visible nuclei are labeled; note that the 1m^2^ nuclei are labeled simply with their quadrant letter (a,b,c,d) in order to conserve space. In general, ectoblast cells form a cap on the surface of the embryo, sitting atop the large yolky macromeres; in early embryos, all of the ectoblast nuclei thus are found within a short focal distance. As epiboly proceeds, the ectoblast cap spreads out over the macromeres until it completely encloses them; at all times, ectodermal nuclei are distributed more or less evenly throughout this superficial cell sheet. The mesoblast daughters of 4d meanwhile spread out underneath the ectoblast sheet; below this layer remain the nuclei of the 3ABC macromeres and their mitotically quiescent daughters, which also tend to be localized toward the embryo's surface. The embryo is about 160 microns in diameter.

Compared to the first quartet micromeres, cell cycle lengths are more nearly uniform among their four apical daughters: the 1abc^1^ cycles last about 145 minutes (1a^1^ tending to divide first), and the 1d^1^ cycle about 155 minutes. Due to the delay in its formation, however, 1d^1^ divides about 45 minutes after its neighbors (450 minutes after first cleavage; **Figures 8** **,** **9**). All four 1m^1^ cells divide dexiotropically. The 1abc^1^ cells each divide with a slight asymmetry, forming a smaller ‘apical cell’ (1abc^11^) and a larger ‘basal cell’ (1abc^12^). As previously noted [12], 1d^1^ divides with a more pronounced asymmetry which, compared to the other three divisions, is reversed in polarity: the apical daughter cell 1d^11^ is about the same size as the three other apical cells, while the basal cell 1d^12^ does not appear to be much bigger than the tiny turret cells. Within each quadrant, the dexiotropic division of 1m^1^ places apical and basal sister cells roughly forty-five degrees out of register with each other around the egg axis. With reference to the secondary axis (approximately defined at this stage by the ‘dorsal’ 3D macromere) the basal cells 1m^12^ are positioned on dorsal, ventral, and lateral radii of the head rudiment. Their apical sisters occupy interradial positions (1cd^11^ right- and left-dorsal, 1ab^11^ left- and right-ventral), in register with the turret cells (1m^2^).

**Figure 9 pone-0005506-g009:**
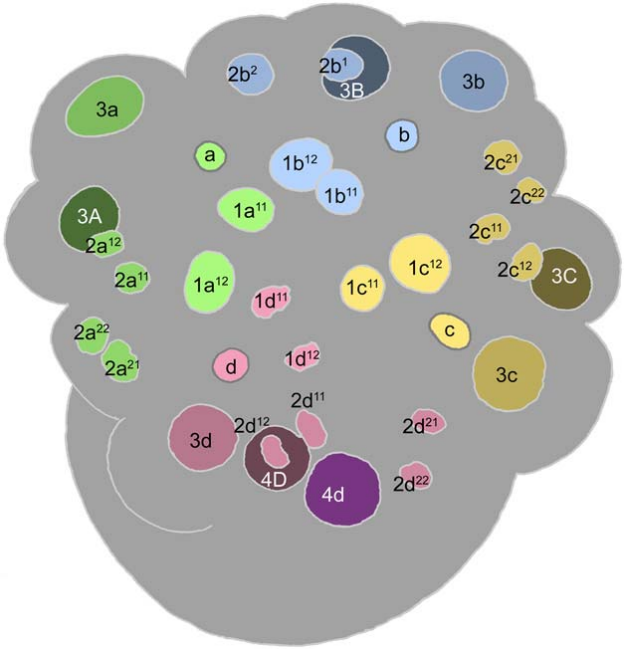
35-cell embryo, 450 minutes. The 1d^1^ cell has just divided (later than its within-tier cousins). The daughter cells of the second-quartet micromeres 2abc have just divided, while both daughters of 2b are lagging behind in prometaphase. The 3cd and 4d cells are entering prophase. This embryo is also shown in Figure 24, with nuclei color-coded according to position on the secondary axis.

**Figure 10 pone-0005506-g010:**
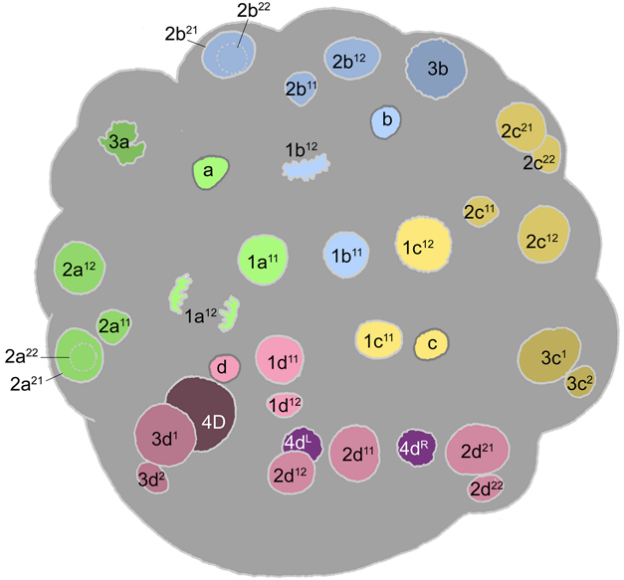
41-cell embryo, 510 minutes. The 1abc^12^ cells are at different stages of mitosis, and the 3a and 3b cells are respectively in prometaphase and prophase. The 4d cell has just divided.

The 1abc^12^ cells are the next first-quartet derivatives to divide, between 515 and 555 minutes after first cleavage (**Figure 10**). Invariably the 1a^12^ cell divides first. These divisions bring about the first sign of dorsoventral asymmetry among the 1abc lineages. According to the rule of alternating division chirality that predominates in early cleavage, all three cells should divide laeotropically. 1a^12^ tends to follow this rule, but the contralateral 1c^12^ cell tends to divide dexiotropically; the division of 1b^12^ is roughly longitudinal. Thus, 1a^122^ and 1c^122^ both tend to be shifted ventrally relative to their sisters.

**Figure 11 pone-0005506-g011:**
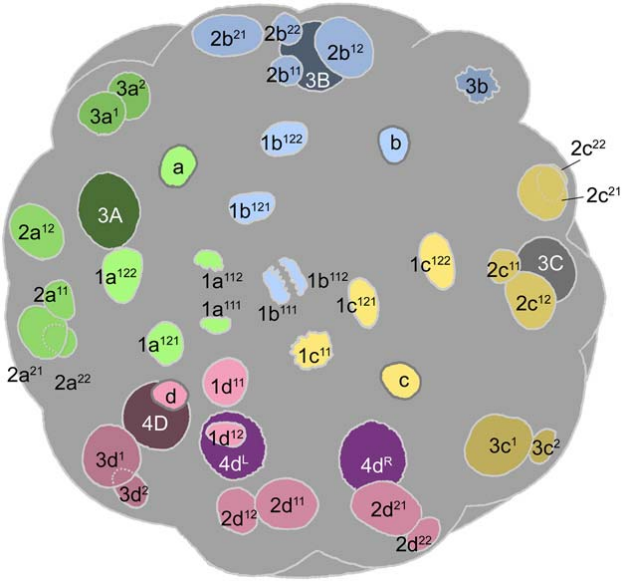
45-cell embryo, 540 minutes. The 1abc^12^ cells have finished dividing. Note the bilaterally symmetric arrangement of 1ac^12^ daughter cells. The 1abc^11^ cells are in slightly asynchronous mitoses. 3a has divided, and 3b is in metaphase.

About 30 minutes after the division of 1abc^12^, the apical cells 1abc^11^ begin to divide (**Figure 11**). The left- and right-ventral 1a^11^ and 1b^11^ cells divide more or less in synchrony, followed by 1c^11^ about 20 minutes later. The 1d^11^ cell divides after an additional delay of about 65 minutes. The cell cycles of 1ab^11^, 1c^11^, and 1d^11^ are respectively estimated as 160, 180, and 200 minutes long. All four cells divide laeotropically; as a result, the ‘apical rosette cells’ 1m^111^ end up in approximately radial positions around the animal pole, with the ‘peripheral rosette cells’ 1m^112^ approximately interradial like their mothers. This arrangement is not initially evident due to close spacing of the 1m^11^ daughter nuclei (**Figures 11** **,** **12** **,** **13**), but is consistently seen at later stages (**Figures 14** **,** **15** **,** **16** **,** **17** **,** **18, 18** **,** **20** **,** **21**). None of these cells were observed to divide again (cell cycles>370 minutes).

**Figure 12 pone-0005506-g012:**
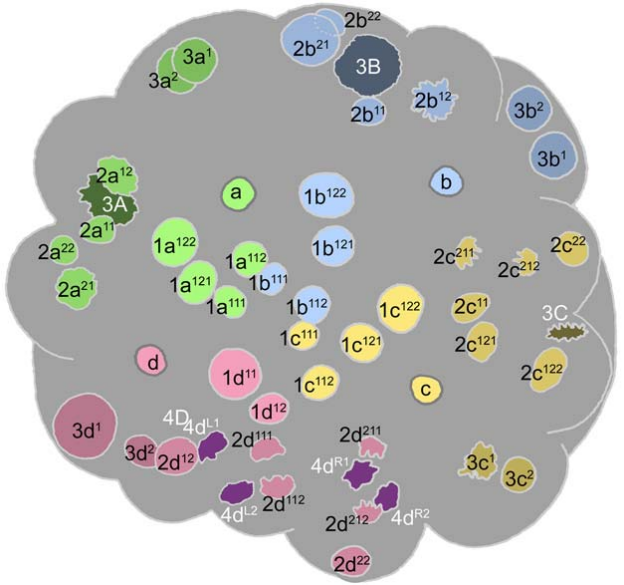
54-cell embryo, 635 minutes. 2c^12^ and 2c^21^ have just divided; the corresponding cells in the 2d lineage are in telophase, and those in the 2a lineage are between metaphase and anaphase. 2b^12^ is in prometaphase, and 2b^21^ has not entered mitosis. Mitoses are beginning in the 3ABC cells, while the left and right daughters of 4d have just divided. The 3c^1^ cell is in metaphase. The outline of the 4D cell is not shown in this drawing.

**Figure 13 pone-0005506-g013:**
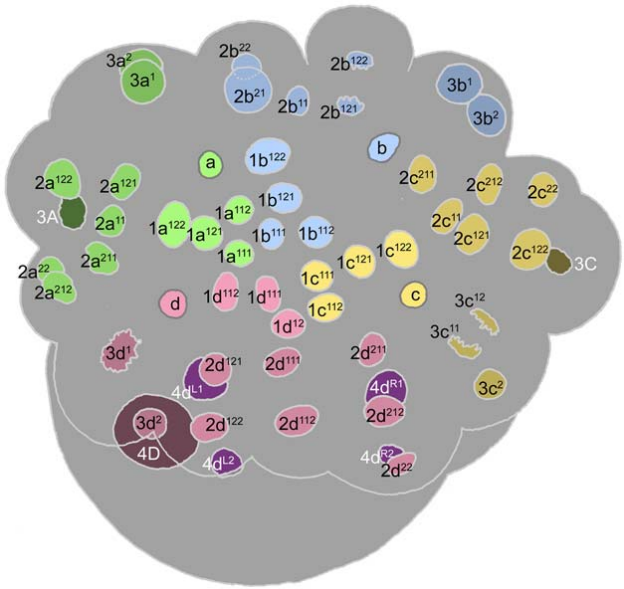
59-cell embryo, 635 minutes. Both 3c^1^ and 3d^1^ are in mitosis; the granddaughters of 2acd have completed their divisions, while 2b^12^ is just completing its division and 2b^21^ is still in interphase. The daughters of 4d^LR^ have reformed interphase nuclei, showing the same pattern of asymmetric division on both sides.

**Figure 14 pone-0005506-g014:**
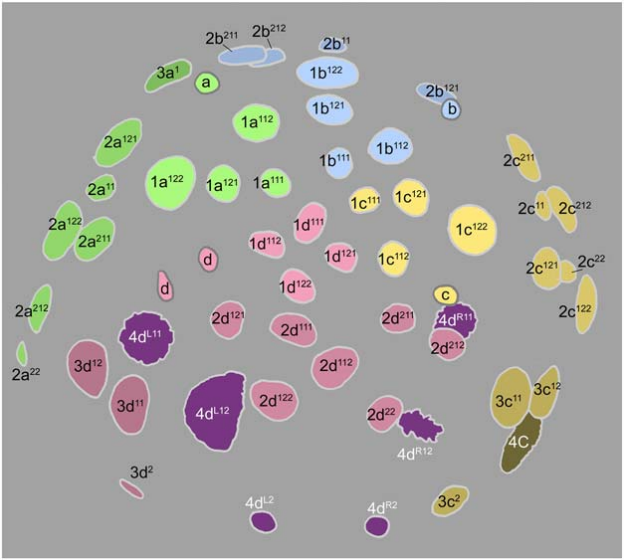
67-cell embryo, 670 minutes. The 4d^LR1^ cells have just divided; the posterior daughters have irregularly shaped nuclei. With the onset of epiboly, the ectoblast has become hemispherical, as reflected by the foreshortening of the 2a and 2c derivatives in this slightly dorsal view.

**Figure 15 pone-0005506-g015:**
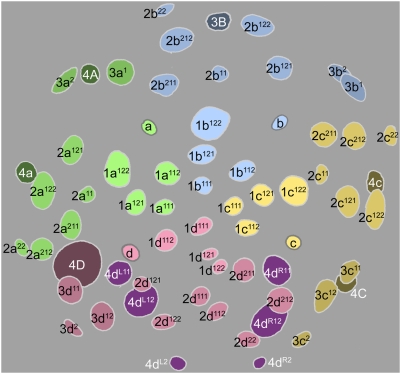
67-cell embryo, 730 minutes. This animal pole view shows the characteristic arrangement of second-quartet nuclei.

**Figure 16 pone-0005506-g016:**
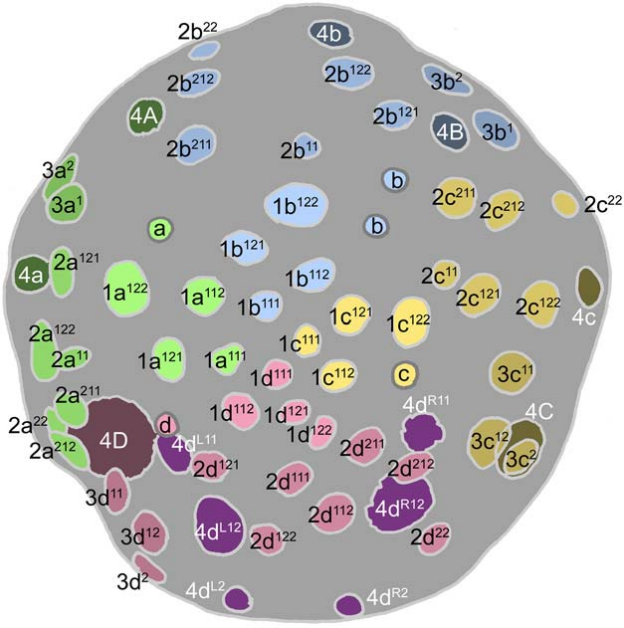
67-cell embryo, 760 min. Atypically, this embryo exhibits daughter nuclei of both 1b^2^ and 1d^12^.

**Figure 17 pone-0005506-g017:**
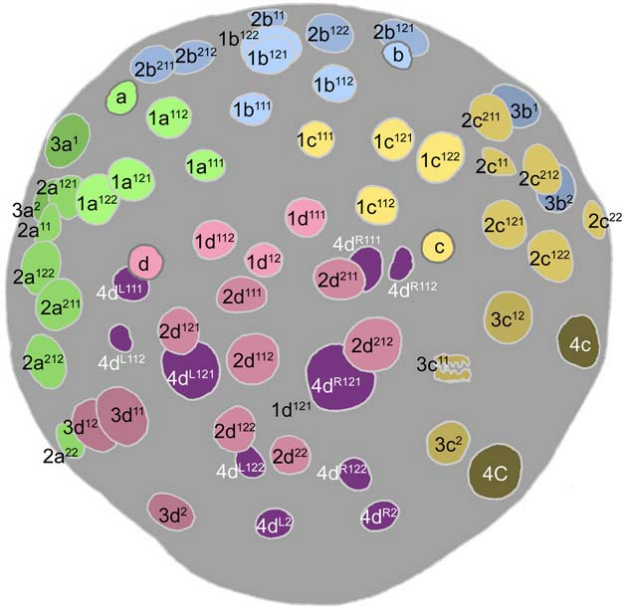
71-cell embryo; 800 minutes. Dorsal view. The 3c^11^ cell is in anaphase; two pairs of 4d derivatives have divided during the preceding hour.

**Figure 18 pone-0005506-g018:**
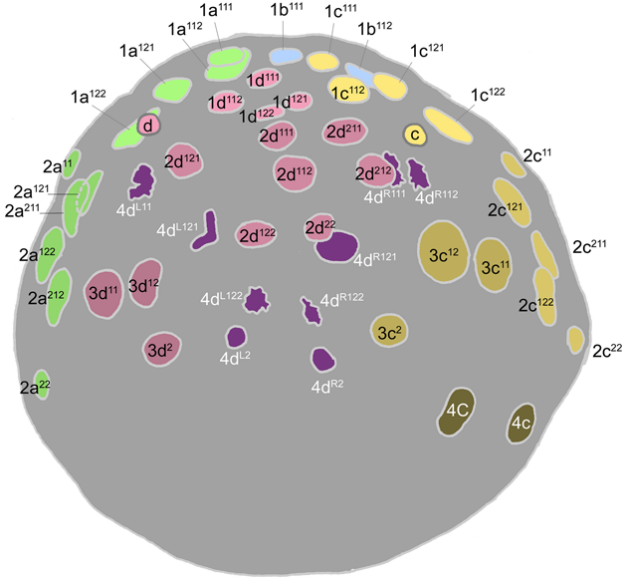
70-cell embryo; 811 minutes. Dorsal view, showing extent of epiboly.

**Figure 19 pone-0005506-g019:**
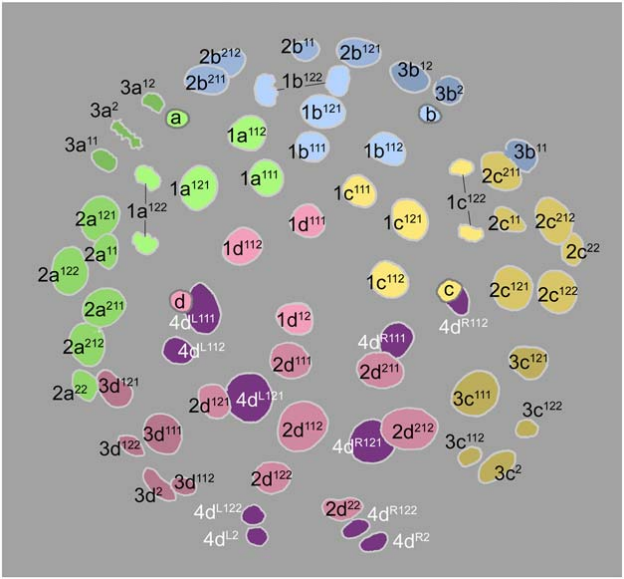
80-cell embryo; 890 minutes. The 1abc^122^ cells have just divided. Both pairs of upper cells in the 3cd lineages have completed their unequal, equatorial divisions; the upper cells of the 3ab lineages have just divided meridionally, and 3a^2^ is in metaphase.

**Figure 20 pone-0005506-g020:**
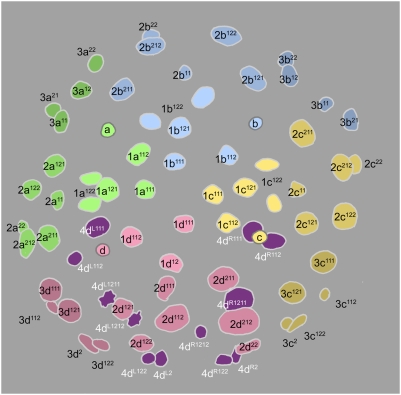
84-cell embryo, 955 minutes. The 4d^LR121^ cells have just divided (4d^L121^ daughters are still in telophase). This embryo is also shown in Figure 24, with nuclei color-coded according to position on the secondary axis.

**Figure 21:**
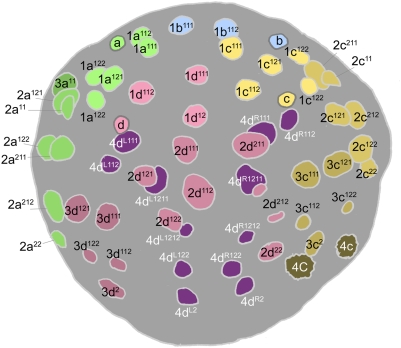
84-cell embryo, 955 minutes. This dorsal view shows how second and third quartet clones are increasingly extended along the animal-vegetal axis. The 2d^212^ cell is dividing. The asymmetry of both 4d^LR121^ is apparent, and the large 4d^LR1211^ nucleus is irregularly shaped.

Prolonged cell cycles also occur in the daughters of 1abc^12^. In particular, the inner cells 1abc^121^ were never observed to divide (cell cycles>420 min). Their sisters 1abc^122^ divide 860–905 minutes after first cleavage (cell cycles≈340–360 min) (**Figure 19**). Unlike all previous divisions in the first quartet, these divisions appeared to be more or less perfectly transverse in all three cells. The 1b^122^ cell is consistently the first to divide, preceding 1ac^122^ by 10–20 minutes.

### Second quartet (Figure 5)

The second quartet micromeres (2m) are formed by laeotropic division of the macromeres 1M. This round of divisions is slightly asynchronous, led by 1CD. The 2m cells are uniform in size, and perceptibly bigger than the 1abc cells. The second quartet cells divide at the same time as their macromere sisters, in the transition from the 16-cell to the 24-cell stage. Division of the 2m cells occurs with a slight asynchrony matching the timing of their birth; these divisions are all equal and dexiotropic, but nearly transverse [12] (**Figure 8**).

The second round of division in the second quartet takes place 445–495 minutes after first cleavage (**Figure 9**). The daughter cells of each second quartet micromere divide in approximate synchrony with each other. Between quadrants, however, a novel timing difference now appears. 2d^1^ and 2d^2^ always divide a few minutes ahead of the 2ac daughter cells, and 2b^1^ and 2b^2^ divide only after another 15–20 minutes. In the 2a, 2b, and 2c lineages, both sister cells divide dexiotropically and very unequally, but with opposite polarities: the upper 2abc^1^ cells bud off a small cell (2abc^11^) toward the animal pole, while the lower 2abc^2^ cells bud off a small cell (2abc^22^) toward the vegetal pole. In the D quadrant, the division geometry is different [12]. Both cells divide dexiotropically as in the other quadrants. The lower cell 2d^2^ divides very unequally like 2abc^2^, but the division of the upper cell produces daughters (2d^11^ and 2abc^12^) that are roughly equal in size (not shown, but note nuclei in **Figures 10** **,** **11**).

The small cells 2abc^11^ and 2m^22^ have small, condensed nuclei and display very long cell cycles (>475 min), similar to the turret cells and 1d^12^ in the first quartet. In contrast to the small 2abc^11^ cells, 2d^11^ divides approximately in synchrony with its equal-sized sister (cell cycles 165–175 minutes) (**Figure 12**). All the other large cells of the second quartet also divide around the same time. The wave of mitoses is consistently begun by 2c^12^. All of these divisions are equal and approximately longitudinal. As their precursors did in the last round of divisions, the 2b^12^ and 2b^21^ cells both lag behind their counterparts in other quadrants, this time by 15–40 minutes (**Figure 13**). After this series of divisions is complete the 2a, 2b, and 2c clones each number six cells, and 2d has formed seven. The arrangement of 2abc derivatives has at this stage become bilaterally symmetric. This is due to a second deviation of sister cell positioning from the rule of spiral cleavage: the upper division products in the A and C quadrants are all shifted ventrally with respect to their sisters, while in the B quadrant the upper cells are displaced to each side (**Figures 12** **,** **13** **,** **15** **,** **16** **,** **20**). In the 2d clone, the six latest-born nuclei form a unique pattern consisting of three laeotropically skewed columns; as development proceeds, the lower-left nucleus in this array (2d^122^) tends to be displaced away from its sister toward the vegetal pole (**Figures 14** **,** **16** **–** [Fig pone-0005506-g017] [Fig pone-0005506-g018] [Fig pone-0005506-g019] [Fig pone-0005506-g020] **21**).

### Third quartet (Figure 6)

The third quartet micromeres are formed by the dexiotropic division of the 2 M macromeres. The 3d cell is always the first to form (315–330 minutes after first cleavage), and the others follow within five minutes. The third quartet micromeres are situated interradially, nestled between the 2m clones; thus, the quartet consists of two dorsolateral cells (3cd) and two ventrolateral cells (3ab) (**Figures 8** **,** **9**). A distinct bilateral symmetry is immediately observed in the third quartet's collective division pattern [12], and no rotational symmetry about the egg axis is ever apparent. The first round of divisions is strikingly asynchronous, led by the simultaneous division of the dorsal cells 3c and 3d (480–500 minutes after first cleavage) (**Figure 10**). These divisions are longitudinal and very unequal, budding off small vegetal daughter cells (3cd^2^) with very small, condensed nuclei and long (>450 min) cell cycles. Approximately an hour after this division, the ventral cells 3a and 3b divide in near synchrony with each other (**Figure 11**). These divisions are also longitudinal, but equal (**Figure 12**).

The 3cd^1^ cells are next to divide, beginning with 3c^1^ (635–650 minutes after first cleavage) and followed 5–15 minutes later by 3d^1^ (**Figures 12** **,** **13**). Each cell divides transversely and equally. This results in the formation of two bilateral pairs of cells: the more dorsal (medial) cells are called 3cd^11^ and their ventral (lateral) sisters are 3cd^12^ (**Figures 14** **,** **15**).

3cd^11^ and 3cd^12^ divide again about 3.5 hours later, before any other divisions in the third quartet have occurred. All four cells divide in the manner of 3cd, the lower daughter cells again being very small (**Figures 19** **–** [Fig pone-0005506-g020] **21**). This round of divisions is led by 3c^11^ around 800–830 minutes after first cleavage (**Figure 17**); the subsequent division timing of 3d^11^ and the 3cd^12^ pair was not accurately determined, but all divisions were completed within the next 50 minutes.

The daughters of 3ab finally begin to divide around 890 minutes after first cleavage, nearly six hours after they were formed (**Figure 19**). In the few cases examined, the upper cells were seen to divide first. The division is transverse and equal in all four cells.

### Fourth quartet/Mesentoblast 4d (Figure 7)

The 3D cell divides asymmetrically about 430 minutes after first cleavage, producing the large mesentoblast cell 4d and the very large vegetal cell 4D. The 4d cell is budded laeotropically toward the micromere cap, and occupies a radial position in line with the middle of the 2d clone (**Figure 9**). The 4abc cells are formed much later (635–675 minutes after first cleavage) in roughly transverse laeotropic divisions (**Figures 15** **,** **16**). Neither the 4abc cells nor any of the remaining macromeres were ever seen to divide again (cell cycles>300 min). The 4d clone, by contrast, proliferates much faster than any other lineage during the next eight hours of development.

The division of 4d is equal and transverse, forming left and right daughter cells 4d^L^ and 4d^R^ (**Figure 10**) This division occurs around 490–510 minutes after first cleavage. Both left and right cells divide again in close synchrony 615–630 minutes after first cleavage (**Figure 12**). Both divisions are unequal [12], the lower cells 4d^LR2^ having much smaller nuclei than the upper cells 4d^LR1^ (**Figure 13**). Similar to the small, mitotically quiescent ectodermal cells with condensed nuclei, the lower cells were never seen to divide (cell cycle length>320 minutes). According to Conklin these small cells (termed E^1^ and E^2^) are the precursors of the hindgut in *Crepidula* [6].

The upper cells, representing the putative mesoblast (Me^1^ and Me^2^ in *Crepidula*) divide again after only about an hour (675–700 minutes after first cleavage). These divisions are synchronous and oriented at bilaterally symmetric angles to the egg axis. The more ‘ventral’ (or ‘anterior’) daughter cells 4d^LR11^ have somewhat smaller nuclei than their sisters 4d^LR12^ (**Figure 15**).

Both pairs of 4d^LR1^ daughter cells divide again very shortly (**Figures 17** **–** [Fig pone-0005506-g018] **19**). In most cases the posterior pair (4d^LR12^) was seen to have completely divided before the onset of mitosis in the anterior pair. The reverse was seen in a few cases; in every case division synchrony was closer within than between bilateral cell pairs. The 4d^LR12^ pair divide along axes that are again roughly parallel to the median plane, though some variation is seen. A slight left–right asynchrony was consistently seen, as the right-sided 4d^R12^ generally had completed its division while its neighbor was in anaphase. Both 4d^L12^ and 4d^R12^ give rise to a more anterior cell with a larger nucleus (cell 4d^LR121^) and a more posterior cell with a much smaller nucleus (cell 4d^LR122^). The timing of these divisions ranged from 740 to 810 minutes after first cleavage. The anterior pair 4d^LR11^ always appeared to divide equally, along oblique axes that were roughly perpendicular to those of the previous division.

Finally, the large anterior nuclei (4d^LR121^) produced by the last anteroposterior division divide again within the next 1.5 hour (**Figures 20** **,** **21**). Once again, the anterior daughter cells 4d^LR1211^ have much larger nuclei than their sisters. The 4d^LR1211^ cells have previously been reported to undergo two more stem-cell-like divisions over the next fourteen hours, budding two more pairs of small, slow-dividing cells toward the midline; in the meantime, the 4d^LR112^ lineages likewise give rise to three cells each [24].

## Discussion

### I. Overview of developmental stages

**Figure 22:**
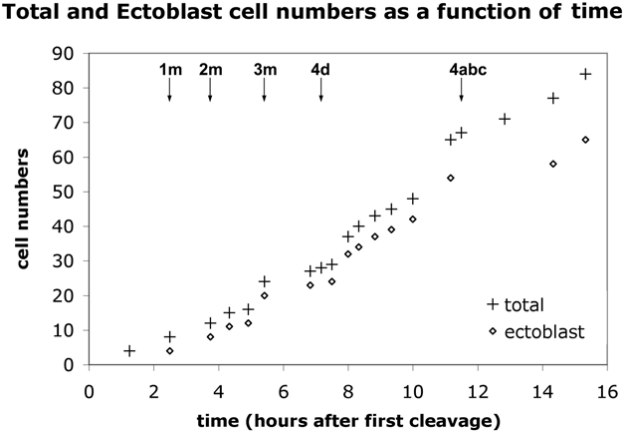
Cell numbers charted over time. Cell numbers increase more or less linearly over time in the whole embryo and in the ectoblast. Micromere births are noted at top.

The cell lineage tables presented here show the timing of all divisions from the four-cell to the 84-cell stage. Proliferation rates are heterogeneous among micromere lineages but tend to decrease over the first three or four cell cycles; thus, total cell number increases almost linearly, with irregular fits and starts (**Figure 22**). The 28-cell stage is marked by the birth of 4d [12], about seven hours after first cleavage. Over the next five hours, 37 more divisions occur in such close succession that few persistent stages can be defined in terms of total cell number. Divisions during this five-hour period include only the 1d^1^ cell and the 1m^11^ tier of the first quartet. In the second quartet, two rounds of division during this period bring the number of 2m derivatives to 25. The second round of 2m divisions (division of 2d^11^, 2m^12^, 2m^21^) is spread over an hour or more, an interval that also includes division of the bilateral 3cd^1^ and 4d^LR^ pairs, as well as the 3ABC macromeres. Finally, division of the 4d^LR1^ pair brings the embryo to 65 cells. Two more rounds of division in the 4d lineage then mark well-defined 67-cell and 71-cell stages, collectively spanning a three-hour period during which the fifty-four ectoblast cells do not divide. The next cells to enter mitosis are the four 3cd^1^ derivatives and the two 4d^LR121^ cells. Shortly thereafter, division of 3ab^1^, 3ab^2^, and 1abc^122^ bring the total cell count to 84. The latest dividing cells among this group were consistently 3ab^2^. Less than an hour later, continuing proliferation in the 2d lineage obscured cell identities among 2d, 3cd and 4d derivatives.

During the last five hours of development followed here, the ectoblast begins to spread out over the macromeres. While this process of epiboly has still not been followed to completion, it seemed in this study to begin 10–11 hours after first cleavage (compare **Figures 11** **–** [Fig pone-0005506-g012] **13** with **Figures 14** **–** [Fig pone-0005506-g015] [Fig pone-0005506-g016] [Fig pone-0005506-g017] [Fig pone-0005506-g018] [Fig pone-0005506-g019] [Fig pone-0005506-g020] **21**) After this stage, nuclei of the first quartet are widely spaced, and distributed in a more regular and stereotyped pattern than before. Another descriptive study reported a similarly abrupt flattening and spreading of the micromere cap at a stage (14 hours at 20°C) coinciding with division of the 3C cell [35]. The present study shows that this stage also corresponds closely with the divisions of 2d^11^, 2m^12^, 2m^21^, 3cd^1^, 3AB, and 4d^LR1^, and that the progress of epiboly over the next three or four hours coincides with mitotic quiescence of the ectoblast.

### II. Reproducibility of cleavage pattern

Up to the 84-cell stage, spatial relationships of nuclei are reproducible enough that the trained eye may identify each cell unequivocally in almost any embryo. Recognition of cell patterns is greatly facilitated by a set of highly unequal divisions whose smaller daughter cells (1m^2^, 1d^12^, 2abc^11^, 2d^22^, and 3cd^2^) undergo prolonged cell cycle arrest and have small, condensed nuclei. Occasionally one of the 1m^2^ cells and/or 1d^12^ was found to have divided several hours earlier than usual (**Figures 14** **–** [Fig pone-0005506-g015] **16** **,** **18**), but daughter cells of 2abc^11^, 2m^22^, or 3cd^2^ were never observed.

One might expect that due to metabolic noise, cell division timing should become progressively less predictable as development proceeds; such time-dependent decorrelation has indeed been noted in an early study of spiralian cell lineage [37]. Even at the latest stages examined in this study, however, the timing of at least some cell divisions among embryos within a brood was highly predictable. For example, between 855 and 905 minutes after first cleavage, only two embryos with an interphase 1abc^122^ nucleus occurred among nine samples (three embryos per sample) that also included the respective cell's postmitotic daughters. Such persistent correlation of division timing among distantly related cells might reflect intercellular coupling of cell cycle progression. Note that this analysis excluded individuals deviating from within-brood synchrony at the earliest stages (see Material and Methods).

It is noted that the overall rate of development observed in this study (which was consistent over a period of over three years) significantly exceeded that reported in a previous study of meiosis and early cleavage [32]. The cause of this discrepancy is unexplained, but might reflect differences among wild snail populations. In general, timing variation between broods from a given population is expected to be much greater than within a single brood, even at early stages. This is suggested by prior observations of between-brood variation in the timing of 3D signaling [16], [38].

### III. Between-tier differentiation of cell cycle rates: preliminary comparisons within Caenogastropoda

To begin understanding the evolution of embryonic cell lineage in *Ilyanassa,* some elements of cell division pattern can be compared with related taxa. *Ilyanassa* belongs to the enormously successful superorder Caenogastropoda, whose monophyly is robustly supported [39]. Cleavage has been described to a comparable extent in two other caenogastropod genera, *Bithynia* [27], and *Crepidula* [6], [11], [26]. Although the relationship of these two genera and *Ilyanassa* is not yet well resolved by molecular phylogenetic methods [39], a comparison of cell lineage parameters is nonetheless illuminating.

**Figure 23:**
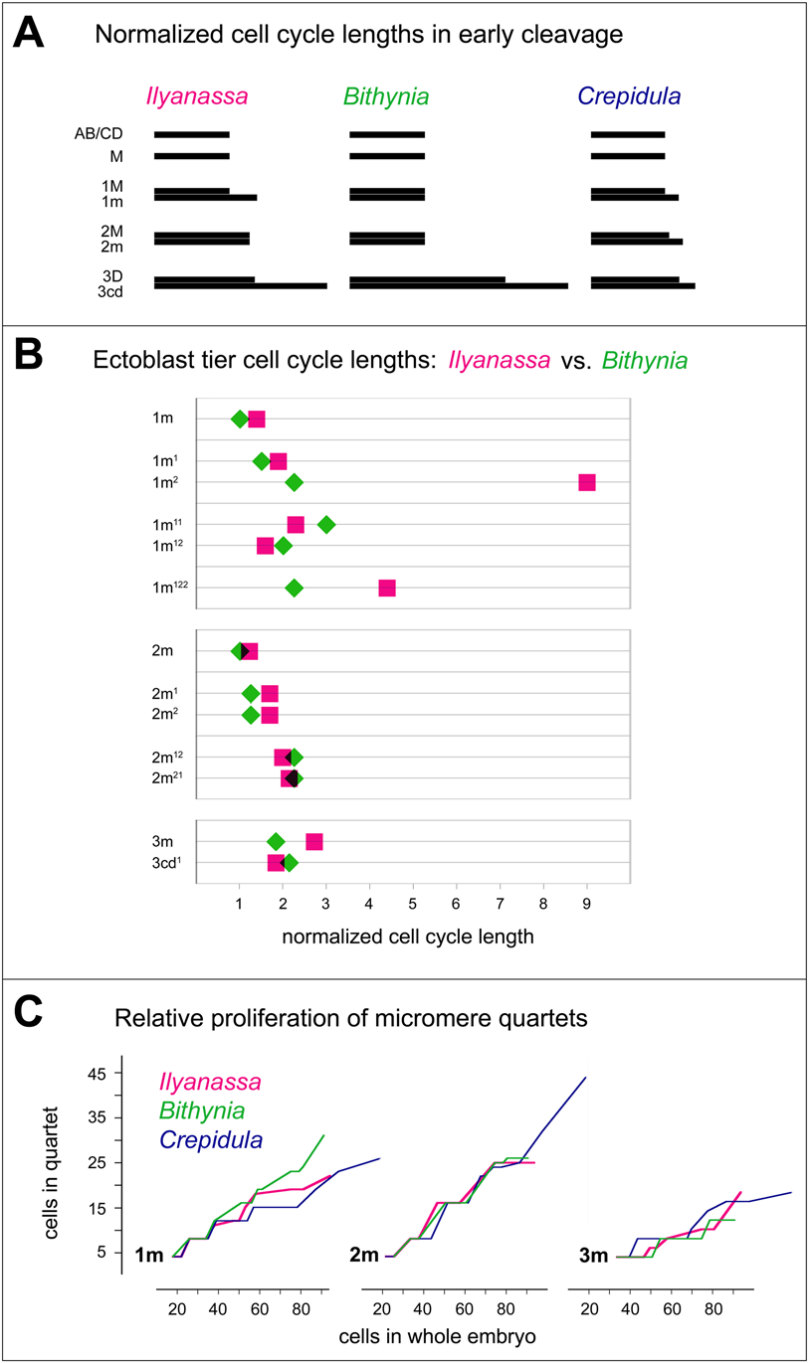
Between-tier cell cycle differentiation in *Ilyanassa* and two other caenogastropods. (A) Comparison of cell cycle lengths during early cleavage, normalized to the species-specific length of cell cycles in AB/CD, M, 1M. *Ilyanassa* shows the most complex pattern of betwen-tier differentiation. (B) Normalized cell cycle lengths in later-formed ectoblast tiers of *Ilyanassa* and *Bithynia.* See text for comments. (C) Relative proliferation rates of micromere quartets compared between caenogastropod genera. In order to compare *Ilyanassa* and *Bithynia* with *Crepidula* (for which later chronological data are lacking), quartet cell numbers are plotted against cell numbers in the whole embryo; values in the three charts are consequently interdependent, and also dependent on cell proliferation in the 3M lineages (not shown).

One aspect of cell lineage that can be meaningfully compared between spiralian taxa is the relative timing of mitosis between cell tiers [40]. In gastropod species in which cleavages have been timed, all of the cells AB/CD, M, 1M exhibit a more or less uniform cell cycle length characteristic of the species. By normalizing to the common cell cycle length of these early macromeres, tier-specific cell cycles can be reasonably compared between taxa. Comparing the early cell cycles of *Ilyanassa*, *Bithynia*, and *Crepidula* shows that each genus has a unique pattern of tier-specific cell cycle lengths (**Figure 23A**). *Ilyanassa* exhibits the most complex pattern, with neither a single abrupt transition to longer cell cycles as in *Bithynia*, nor gradually increasing cell cycle lengths as in *Crepidula.* Extending the comparison of normalized cell cycle lengths between *Ilyanassa* and *Bithynia* suggests several generalities (**Figure 23B**). In each genus, some ectoblast tiers divide faster and others slower. Sister-cell pairs tend to exhibit concordantly shorter or longer cell cycles in a given genus (4/4 instances). In both genera, cell cycle length increases progressively in the second-quartet clones. Two first-quartet tiers (1m^2^ and 1m^122^) have extremely long cell cycles in *Ilyanassa* but not in *Bithynia*. Ignoring these two outliers, the mean normalized cell cycle length is very similar in the two genera (1.8 and 1.9, where the cell cycle length in AB/CD, M, 1M is defined as 1).

Relative proliferation rates of the three micromere quartets can be further compared by using cell numbers in the whole embryo as a yardstick (**Figure 23C**). The only conspicuous difference among the three caenogastropod genera is a relatively high growth rate of the first quartet in *Bithynia.* This disparity, which largely reflects the unusually short cell cycles of 1m^2^ and 1m^122^ in *Bithynia,* becomes increasingly pronounced up to the latest stages examined; if it persists through later development, it might lead to heterochrony in organogenesis, akin to that recently described among basommatophoran pulmonate snails [41].

### IV. Within-tier cell cycle differentiation: earlier departures from radial symmetry in *Ilyanassa* compared to other caenogastropods

Soon after signaling from 3D/4d, the form and timing of cell division begins to differentiate within most ectoblast tiers. Cells within a tier can be considered serially homologous, owing to their synchronous and topologically equivalent origins. The homology of cells within each tier is further supported by the tier-specific localization of numerous, randomly selected RNA species (by contrast, no quadrant-specific RNAs have yet been found) [5]. In the absence of D quadrant specification, the four cells of each tier divide with identical timing and geometry [12]. Within-tier differences in developmental potential and early mitotic behavior are evidently imposed on a homogeneous ground state by two sources. First, the polar lobe exclusively distinguishes the D lineage, and could in principle contribute unique information to the D lineage cell in each tier. Intrinsic control of D quadrant micromere development has been shown only for the 1d cell [12], [21] (see section VII). Second, dorsal and lateral cells (generally in the C and D quadrants, and in some tiers also the A quadrant) are influenced by signaling from 3D/4d [12], [21]. Thus, both lineage and position contribute to within-tier differentiation; both mechanisms are expected to generate differences correlated more or less exactly with cell position along the secondary axis. As described below, most within-tier differences in cell division pattern up to the 80+ cell stage are indeed correlated with dorsoventral cell position.

**Figure 24:**
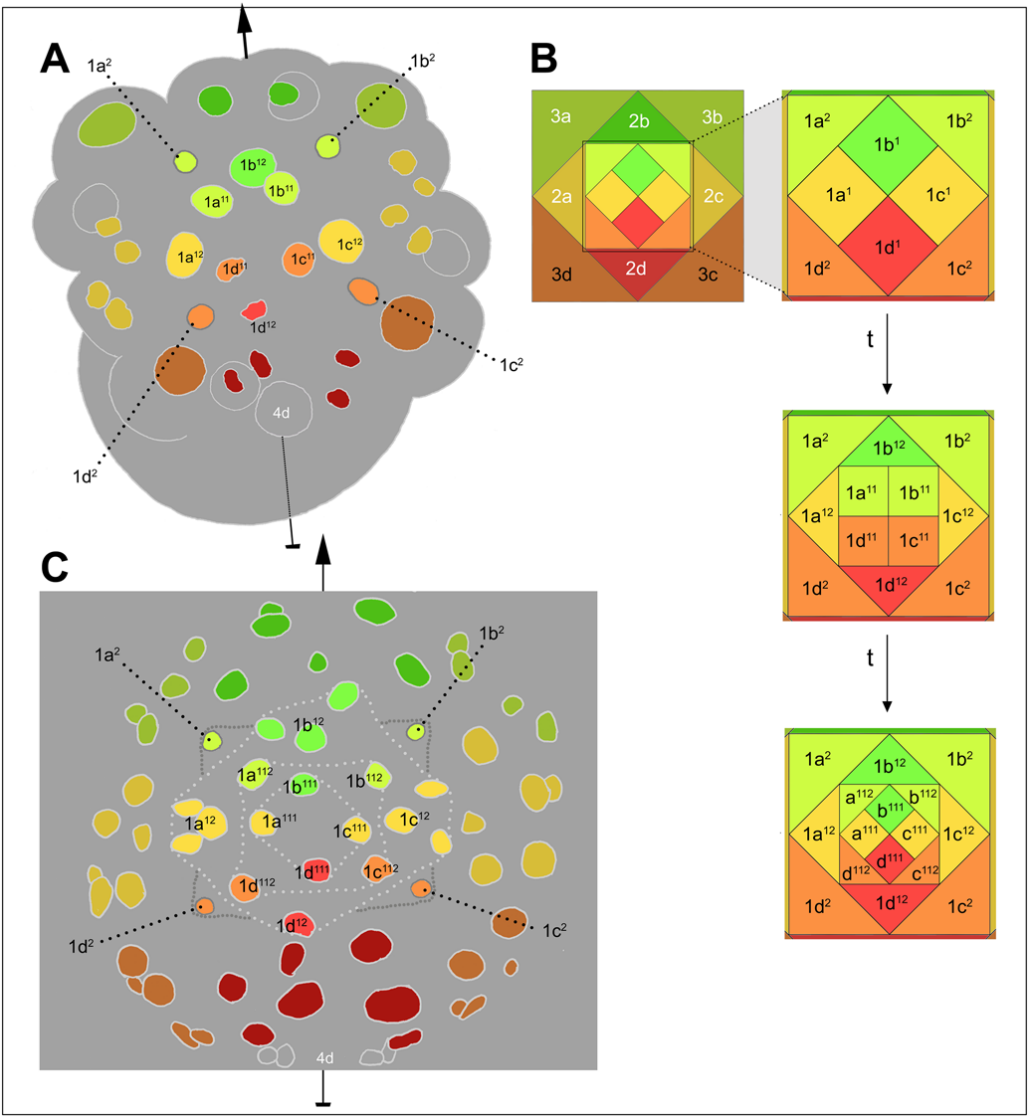
Disposition of micromere sublineages along the secondary axis. (A) 35-cell embryo with nuclei color-coded by position along the secondary axis (indicated with an arrow through the dorsal 4d nucleus and the center of the mid-ventrally fated 2b clone). This stage of development very shortly follows the complete transmission of an ectoblast patterning signal from the 3D/4d cells. (B) Schematic showing ontogeny of ectoblast cell distributions relative to the secondary axis. Upper panels show a slightly earlier stage than (A), when ectoblast patterning is initiated by the 3D macromere (shown realistically in Figure 3B; see also Figure 8). A blow-up of first-quartet cells is shown at right. Subsequent distributions of first-quartet derivatives along the secondary axis are shown in the middle panel (corresponding to embryo in (A) and the lower panel (corresponding to embryo in (C); the coefficient ‘1’ is omitted for 1m^11^ daughters. (C) 84-cell embryo with nuclei color-coded in relation to the secondary axis (arrow). Note that second- and third-quartet clones wholly retain the axial dispositions of their progenitors, as do the 1m^12^ clones; these dispositions accurately predict clonal fates in *Ilyanassa* and other gastropod species (see text).

Cell positions within a tier are remarkably well defined in relation to the secondary axis. During the period when this axis is inductively patterned by the 3D macromere and/or its daughter 4d, the 1m^1^ and 2m cells occupy radial positions (dorsal, lateral, ventral) while the 1m^2^ and 3m cells occupy interradial positions (dorsolateral, ventrolateral) (**Figure 24A**; see also **Figure 3B** **and** **Figure 8**). These approximate geometric relationships between clones persist throughout the period of development studied here (with the secondary axis defined by the center of the 4d clone) (**Figure 24B**). Fate maps of *Ilyanassa*, *Crepidula*, and their distant relative *Patella* corroborate these assignments of relative axial position, at least within the bilaterally symmetric regions of the larval snail (head, velum, mouth, foot) [8]–[11]. Further divisions of the 1m^1^ cells generate both radially distributed tiers (1m^111^; 1m^12^) and the interradial 1m^112^ tier (**Figure 24C–F**). While the fates of these sublineages have not mapped by intracellular dye tracing, cell lineage analysis in *Crepidula* has shown them to make predictable contributions to larval and adult ectoderm [6], and their initial and ultimate axial dispositions are conserved outside of caenogastropods [7].

**Figure 25:**
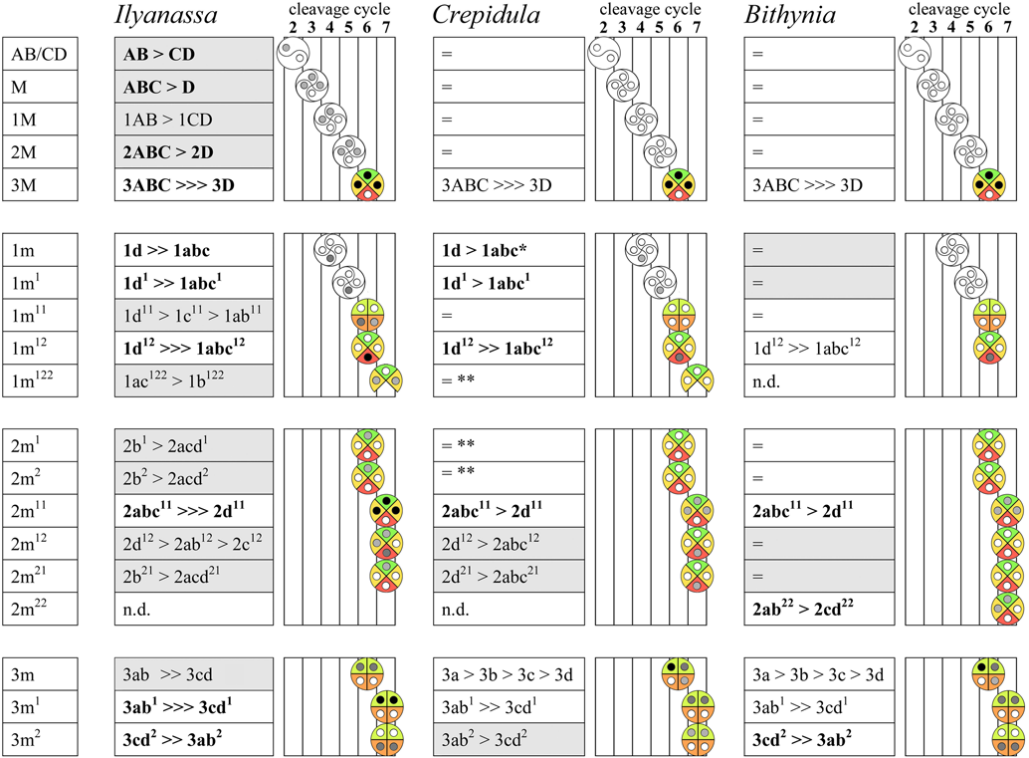
Within-tier cell cycle differentiation in three caenogastropods. Instances of mitotic asynchrony are charted for each tier that exhibits within-tier asynchrony in at least one of the three compared genera. The magnitude of asynchrony is indicated as in the following examples: [AB>>>CD]: AB enters mitosis after the daughters of CD have entered mitosis. [AB≫CD]: AB enters mitosis after division of CD, but before (or simultaneous with) the first division of a CD daughter. [AB>CD]: AB enters mitosis shortly after CD (mitoses overlap, or nearly so). [ = ]: Cell cycles are of equal length. Bold type indicates cases in which a longer cell cycle is clearly correlated with lower cytoplasmic volume (the inverse of this relationship was never observed within a tier). Gray shaded boxes indicate division orders that occur uniquely in one genus. Tier diagrams represent division order by gray values of cartoon nuclei; axial dispositions of cell fates are shown and color-coded as in Figure 24B. * Conklin (1897) makes no mention of asynchrony in the 1m tier, and indeed shows these cells dividing in approximate synchrony in one embryo; however, he states in a subsequent paper (1902) that 1d predictably divides later than 1abc. ** Conklin (1897) shows a slight asynchrony within the 2m^1^ and 2m^2^ tiers in his Figures 26–28, and within the 1abc^122^ group in his Figure 49, but does not mention any predictable asynchrony for either case.

Within-tier division asynchronies in *Ilyanassa, Crepidula,* and *Bithynia* are shown in **Figure 25**. Such division asynchrony is most prevalent in *Ilyanassa*, and least prevalent in *Bithynia.* After the birth of 3D, thirteen of fourteen examined cell tiers divide asynchronously in *Ilyanassa*. Only six of these tiers are reported to divide asynchronously in the same order in *Crepidula*. In each of these six cases, the magnitude of division asynchrony is greater in *Ilyanassa,* and no tier divides with a more pronounced asynchrony in *Crepidula.* In three cases (2m^12^, 2m^21^, 3m^2^) within-tier asynchronies occur with different order between *Ilyanassa* and *Crepidula.*

While most within-tier cell cycle diversification is correlated with cell position along the secondary axis, division asynchrony may also occur between left- and right-sided cells within a tier. Four subtle but stereotyped bilateral division asynchronies were found in *Ilyanassa* which have never been reported in other gastropod species. Curiously, the right-sided cell divides first in each of these cases. First, the right-dorsal 1c^11^ cell has a shorter cell cycle than its left-sided counterpart 1d^11^. Second, the right-sided 2c^12^ cell, though formed at about the same time as the 2a^12^ and 2a^21^ cells on the left, predictably leads the third round of second-quartet divisions. This asynchrony presages a quantitative difference in the morphogenetic contributions of 2c and 2a: the right-sided trunk ectoderm, formed by 2c, exhibits a higher growth rate during late gastrulation and organogenesis [42]; correspondingly, 2c forms a larger region of the mantle edge than 2a [10], and shell development can be severely disrupted by ablating either 2c or 2d, but not 2a or 2b [19]. Finally, two other right-hand cells in *Ilyanassa* (4d^R12^ and 3c^11^) were also found to divide precociously, suggesting a general right-sided growth advantage in multiple lineages. While none of these bilateral asynchronies have been reported in other gastropods, it is noted that the right-sided and dorsal 2cd^22^ cells in *Bithynia* divide before the left-sided and ventral 2ab^22^ (this division has not been described in *Ilyanassa* or *Crepidula*). Preferential right-sided growth and organogenesis is widespread and likely primitive among gastropods [43], suggesting that *Ilyanassa* has evolved an earlier onset of differential growth along the left-right axis as well as along the secondary axis.

### V. Within-tier differentiation in cell division asymmetry: bigger differences in *Ilyanassa*

The geometry of cell division as well as its timing can vary predictably within cell tiers. Three tiers in *Ilyanassa* were found to exhibit quantitative within-tier differences in the geometric asymmetry of cell division; in each case the difference is precisely correlated with cell position along the secondary axis. Each of these differences is reduced or absent in *Crepidula*, *Bithynia*, or both. One case involves the dexiotropic division of the 1m^1^ cells. In *Ilyanassa*, the 1d^1^ cell divides with an asymmetry that is reversed with respect to the asymmetry of the 1abc^1^ divisions. In *Crepidula,* the 1abc^1^ cells divide as in *Ilyanassa*; however, the asymmetry of the 1d^1^ division is not reversed but is merely reduced compared to 1abc^1^ [26]. No difference in division geometry within the 1m^1^ tier was noted in *Bithynia*.

A second within-tier difference in division asymmetry occurs in *Ilyanassa* when 2d^1^ divides equally and the 2abc^1^ cells all divide very unequally. In *Crepidula* and *Bithynia* this differentiation is present in a reduced (or rudimentary) form: the 2abc^1^ cells divide as in *Ilyanassa*, while 2d^1^ divides with a slight asymmetry. In all three genera, cell cycle lengths of the 2m^11^ and 2m^12^ daughter cells are inversely correlated with inherited cell volume; thus, the 2d^11^ cell inherits a growth advantage which is greatest in *Ilyanassa.* Reduced asymmetry of the 2d^1^ division relative to 2abc^1^ may be an ancestral condition, as it is also reported in basal gastropods [30], [44] as well as in two other molluscan classes [45], [46]; however, all four 2m^1^ cells divide with the same extreme asymmetry in some other gastropod taxa [7], [47], [48], as well as in polyplacophorans [49], [50]. *Ilyanassa* is the only mollusk in which 2d^1^ is reported to divide equally.

Finally, in *Ilyanassa* the 3cd cells undergo highly asymmetric divisions while the 3ab cells divide equally; in *Crepidula,* by contrast, all four cells divide with the same slight asymmetry. *Bithynia* displays a third pattern: 3cd bud off small cells toward the vegetal pole, while 3ab bud small cells toward the animal pole. Both the *Ilyanassa* and *Bithynia* patterns have been reported in non-caenogastropod snails [7], [30], [44], [47], [48]; the *Crepidula* pattern is therefore most likely derived from one of the two others.

### VI. Dorsoventral organization of cell division axes: breaking the golden rule of spiral cleavage

**Figure 26:**
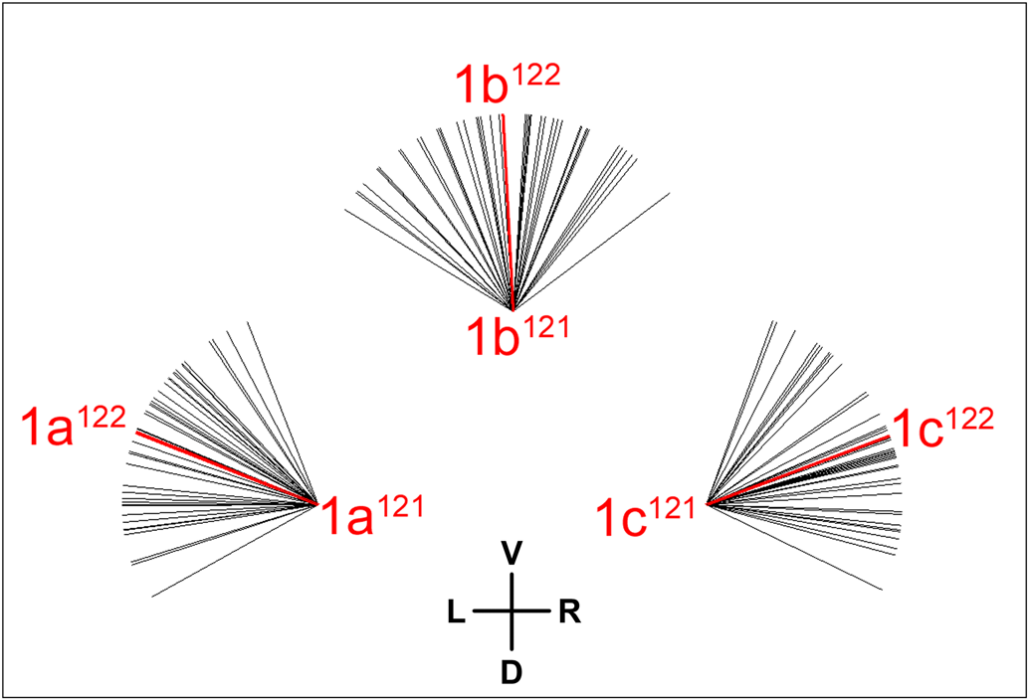
Dorsoventrally polarized division axes in the 1abc^12^ cell group. The orientation of each black line represents the axis between a single pair of 1abc^121^/1abc^122^ sister nuclei (41 embryos total), with the secondary axis of the embryo oriented vertically. Red lines mark mean division axis for each cell. The 1ac^122^ nuclei tend to be positioned more ventrally than their 1ac^121^ sisters.

Another aspect of division geometry is the orientation of division axes relative to the embryo's spatial coordinates. At early stages, oblique division axes generate a chiral, rotationally symmetric pattern; later, division axes within many ectoblast tiers show a collective bilateral symmetry and dorsoventral asymmetry [6], [7]. In general, dorsoventral organization of cell division axes seems to be more prevalent in *Ilyanassa* than in *Crepidula*. For example, the collective division pattern of the 1abc^12^ group exhibits a dorsoventral asymmetry in *Ilyanassa*: although the spatial relationships of daughter nuclei vary considerably, both of the lower daughter nuclei (1ac^122^) are shifted ventrally in most cases (**Figure 26**). In *Crepidula*, the 1abc^12^ cells all divide dexiotropically (surprisingly, as their mothers do too) [6], [26]. In *Bithynia* the 1m^12^ cells are all reported to divide longitudinally.

In several other instances, sister nuclei in related tiers seem to be distributed chirally in *Crepidula* but achirally in *Ilyanassa* (the 3ab daughters) or with a dorsoventral asymmetry in *Ilyanassa* but not in *Crepidula* (collectively, the 2ac^12^ and 2ac^21^ daughters). Comparison of these traits will require morphometric analysis beyond the scope of the present study.

### VII. From fuzzy to rigorous during ontogeny and phylogeny: how early differentiation precedes, then preempts, cell fate decisions

The spatiotemporal geometry of cleavage in mollusks clearly has the potential to contain a great deal of information, but for the most part we do not know its developmental significance. In one instance, however, recent experimental studies in *Ilyanassa* and *Crepidula* have revealed an evolutionarily labile coupling between early differentiation and fate specification. Intriguingly, they suggest a stepwise evolutionary pathway in which accelerated phenotypic differentiation has functionally supplanted a primitive signal in regional specification.

In all examined gastropods, and in two other molluscan classes, part or all of the 1d^12^ clone enters a persistent cell cycle arrest while corresponding cells of the 1abc^12^ lineages proliferate to form definitive head structures [6], [7], [22], [27], [45], [47], [49], [53]. In both *Ilyanassa* and *Crepidula,* this arrest is anticipated by mitotic delays in the 1d, 1d^1^, and 1d^12^ cells [6], [12], [26] (**Figure 25**). In both genera, the 1d cell is born smaller than 1abc, indicating an intrinsically determined difference [12], [26]. Experiments on *Ilyanassa* have shown that the limited size and division rate of 1d depend on polar lobe segregation to the D cell [12]; the polar lobe also prevents 1d from forming an eye [21], evidently by limiting its inherited cytoplasmic volume [35].

Does early differentiation of 1d size and cell cycle length play any role in determining 1d fate in *Crepidula*? As in *Ilyanassa,* the mother cell of 1d inherits a polar lobe at second cleavage in *Crepidula*. By contrast to *Ilyanassa,* however, removal of the polar lobe in *Crepidula* does not appear to endow 1d with eye-forming potential, indicating that a polar lobe-independent mechanism must restrict 1d potential [15]. A cell-extrinsic mechanism of 1d fate restriction undoubtedly operates in more basal gastropods, where the D cell inherits neither a polar lobe nor any other determinant of developmental potential at second cleavage [52]. This ancestral mechanism of 1d fate restriction, which likely operates also in *Crepidula*, appears to have been completely replaced by an earlier, cell-intrinsic mechanism in *Ilyanassa* [21].

Specification of 1d is necessarily tied to that of the dorsal signaling cell 3D; the mechanism of this linkage, however, appears to have changed. In *Ilyanassa*, the early differentiation and fate restriction of 1d is preceded by determination of the D quadrant founder cell as precursor to 3D [12], [21], [51]. Although the polar lobe in *Crepidula* is likewise consistently inherited by the D cell, 3D identity is not specified until the 24-cell stage, as a result of inductive signaling from first-quartet micromeres to one of the 3M macromeres; this inductive mechanism, like the extrinsic specification of 1d, is primitive [50], [52]. The polar lobe in *Crepidula* evidently acts just to bias 3D-inductive signaling to one macromere [15]. The mechanism of this bias is unknown. The early differential behavior of the 1d lineage suggests that 1d itself could be involved in biasing 3D induction. Supporting this notion, selection of one macromere as 3D in the pulmonate *Lymnaea* and the basal gastropod *Patella* depends on the presence of a first-quartet micromere belonging to the same quadrant [55].

Our knowledge of the D lineage in *Crepidula* suggests a fuzzy-logical mode of lineage specification in which phenotypic divergence precedes a definitive cell fate decision. In theory, such an early differentiation event may itself act as a cue to bias cell fate; alternatively, it may depend incidentally on a preceding cue that acts in parallel to bias cell fate. In particular, further study is needed to find out whether an intrinsic quality of 1d biases 3D identity in *Crepidula.* Regardless of its primitive developmental role, such an early differentiation event provides an avenue for the future evolution of accelerated fate restriction. This is illustrated by the case of *Ilyanassa*, where D cell division asymmetry has been exapted to functionally replace a primitive head-patterning signal. Polar lobe segregation too can be regarded as an early cell differentiation event that has, in a gradual or punctuated manner, come to replace inductive specification of 3D during the evolution of *Ilyanassa*.

### VIII. Overview and Prospects

Previous embryological studies of mollusks and other spiralians have shown wide variation in the complexity of early cleavage pattern. Some taxa, including representatives of two of the most basal gastropod clades, display almost no within-tier differentiation up to the latest stages examined (88+ cells)[29], [46], [47]. By contrast, a number of others (including scaphopod and some bivalve mollusks, as well as many annelids) deviate from rotational symmetry to a much greater degree than *Ilyanassa* [43], [44], [55], [56]; at the furthest extreme, the stereotyped cleavage patterns of cephalopods display no trace of spiral geometry [57]. In general, complex patterns of early lineage-specific differentiation seem to have evolved at the bases of several higher-level spiralian clades [50], and transitional states are largely unknown.

The variation documented here among caenogastropods suggests that it may be possible in some cases to reconstruct the evolution of complex early cell division patterns. By focusing on a quantitative aspect of differential behavior among serially homologous cells (i.e., cell cycle length within a tier), one can detect small evolutionary changes in the behavior of single cells; as shown in the case of *Ilyanassa*, many such small changes may summate along body axes to suggest a global evolutionary trend. Extending this analysis in the context of a robust, independent (i.e., molecular) phylogeny may shed light on the evolutionary dynamics of early developmental complexity.
